# Enhanced resistance to the cellulose biosynthetic inhibitors, thaxtomin A and isoxaben in *Arabidopsis thaliana* mutants, also provides specific co-resistance to the auxin transport inhibitor, 1-NPA

**DOI:** 10.1186/1471-2229-13-76

**Published:** 2013-05-03

**Authors:** Robert S Tegg, Sergey N Shabala, Tracey A Cuin, Noel W Davies, Calum R Wilson

**Affiliations:** 1 Tasmanian Institute of Agriculture (TIA), University of Tasmania (UTAS), 13 St John’s Ave, New Town, Tasmania, 7008, Australia; 2 TIA, UTAS, Private Bag 54, Tasmania, Hobart, TAS 7001, Australia; 3 Central Science Laboratory, University of Tasmania, Private Bag 74, Tasmania, Hobart, 7001, Australia

**Keywords:** 1-napthylphthalamic acid - NPA, 2,3,5-Triiodobenzoic acid - TIBA, Thaxtomin A, Isoxaben, Dichlobenil, Cellulose biosynthetic inhibitor, Common scab, Ion fluxes, Plasma membrane

## Abstract

**Background:**

Thaxtomin A (TA) is a phytotoxin produced by plant pathogenic *Streptomyces* spp*.* responsible for potato common scab. TA inhibits cellulose biosynthesis in expanding plant tissues and is essential for disease induction. Auxin treatment of various plant tissues has been repeatedly demonstrated to inhibit TA toxicity and to reduce common scab. This work utilises *Arabidopsis thaliana* mutants with resistance to cellulose biosynthesis inhibitors (CBIs) to investigate the interaction between TA, other CBIs and auxins.

**Results:**

Three CBI resistant *A. thaliana* mutants; *txr1-1* (tolerance to TA), *ixr1-1* (tolerance to isoxaben - IXB) and *KOR1* (cellulose deficiency), showed no altered root growth response to treatment with natural or synthetic auxins, nor with the auxin efflux transport inhibitor 2,3,5-Triiodobenzoic acid (TIBA). However, all mutants had significantly enhanced tolerance to 1-napthylphthalamic acid (NPA), another auxin efflux transport inhibitor, which blocks polar auxin transport at a site distinct from TIBA. NPA tolerance of *txr1-1* and *ixr1-1* was further supported by electrophysiological analysis of net H^+^ fluxes in the mature, but not elongation zone of roots. All three mutants showed increased tolerance to IXB, but only *txr1-1* showed tolerance to TA. No mutant showed enhanced tolerance to a third CBI, dichlobenil (DCB).

**Conclusions:**

We have demonstrated that plant tolerance to TA and IXB, as well as cell wall synthesis modifications in roots, have resulted in specific co-resistance to NPA but not TIBA. This suggests that CBI resistance has an impact on polar auxin efflux transport processes associated with the NPA binding protein. We also show that NPA inhibitory response in roots occurs in the mature root zone but not the elongation zone. Responses of mutants to CBIs indicate a similar, but not identical mode of action of TA and IXB, in contrast to DCB.

## Background

Thaxtomin A (TA) is the major phytotoxin produced by pathogenic *Streptomyces* spp. responsible for common scab, a globally important disease of potato [1]. TA inhibits cellulose biosynthesis in expanding plant tissues and its production is essential for disease induction [2-4]. Based on similarity of symptoms produced, TA is believed to be closely related to other cellulose biosynthesis inhibitors (CBIs) such as isoxaben (IXB) and dichlobenil (DCB) [5]. The linkage of the modes of actions of these compounds has been confirmed with habituation studies to TA, revealing cross-resistance to both IXB and DCB [6], although mechanisms of resistance were not investigated.

The cellular target of TA has not been identified [6,7]. This is in contrast to IXB, where mutant analyses have identified specific cellulose synthase (CesA) complexes (CesA3 and CesA6) from the plasma membrane as toxin targets [8,9]. Putative cellular targets for DCB have also been indirectly identified and include CesA1 or CesA5 and other regulatory proteins [10,11]. A recent study showing similar genes were upregulated following TA and IXB treatments of *A. thaliana* cells suggested a possible linkage in activity between these two CBI’s [12]. An initial interaction between TA and the plant cell membrane, resulting in ion flux signaling has been reported [13], as has induction of programmed cell death [14]. However, little is known about the exact mechanism of cellular toxicity of TA. The TXR1 gene is involved in a cellular transport system and mutations in this gene in *Arabidopsis thaliana txr1-1* lead to a decrease in toxin sensitivity, most likely due to reduced toxin uptake [15].

In prior studies we have demonstrated an inverse association between TA toxicity and auxin or auxin-like compounds [13,16,17]. Foliar treatment of potato plants with auxin and auxin-like compounds has been shown to suppress common scab development [17,18]. Work in our laboratory has provided evidence that the mechanism of disease suppression is due to auxins inhibiting TA toxicity [16,17]. This and other electrophysiological data, whereby an auxin sensitive *A. thaliana* mutant (*ucu2-2 and gi2)* showed increased sensitivity to TA [13] further support the link between auxin and TA toxicity. However, other researchers [12] have questioned the direct causal relationship between TA and auxin itself as they noted very few auxin genes were upregulated in response to TA. Thus, the interaction between auxin and TA remain elusive.

Utilising CBI resistant *A. thaliana* mutants that are well characterized may provide an important resource for delineating and understanding disease resistance pathways and mechanisms of action and interactions [19]. In the case of TA, a resistant mutant *txr1-1* has been described [15] as has an IXB resistant mutant *ixr1-1*[20] and a loss-of function mutant in genes required for cell wall synthesis *KORRIGAN* (*KOR1*) [21,22]. These mutants and others may play a role in delineating similarities and differences in reaction to CBIs and auxinic compounds and serve as useful tools in defining further interactions between TA and associated compounds.

In this work, we investigate the unique interaction of TA toxicity and its amelioration by auxin. We demonstrate that the interaction is not a direct effect of auxin *per se*, but is rather mediated through an interaction with polar auxin transport, associated with the NPA binding protein of the efflux carrier. Further, responses of key mutants indicate a probable commonality in mode of action of both IXB and TA, distinct from DCB which appears to be more distantly related.

## Results

### Mutant screening against various auxin sources

**Figure 1:**
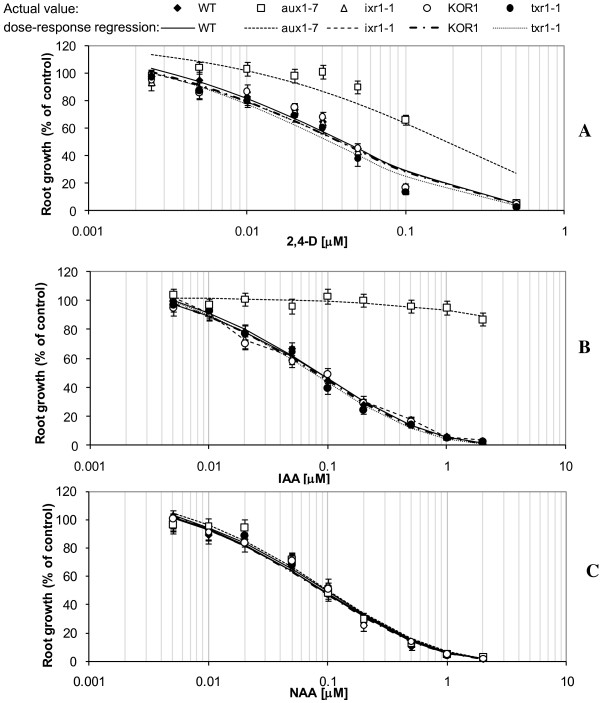
**Root growth suppression of *Arabidopsis *lines in response to auxin treatment.** Wild type and mutant *Arabidopsis thaliana* seedlings were treated for 72 h on medium containing: (**A**) 2,4-D; (**B**) IAA; (**C**) NAA. Individual data points are expressed as mean percentages ± SE (n = 20) of control root growth on medium with no auxin applied.

Consistent with previous studies [23], *aux1-7* had enhanced resistance to both 2,4-D (*P* = 0.0004, *F* = 15.6) and IAA (*P* < 0.0001, *F* = 35.4), but not to NAA (*P* = 0.81, *F* = 0.06), when compared to RG_50_ value of the WT control (Figure 1). The TA resistant line *txr1-1* (*P* = 0.32-0.85, *F* = 0.04-1.02), *ixr1-1* (*P* = 0.78-0.93, *F* = 0.01-0.08) and *KOR1* (*P* = 0.56-0.95, *F* = 0.01-0.56) all showed sensitivity to all three auxins equivalent to the WT control (Figure 1).

### Mutants with altered response to auxin transport inhibitors

**Figure 2:**
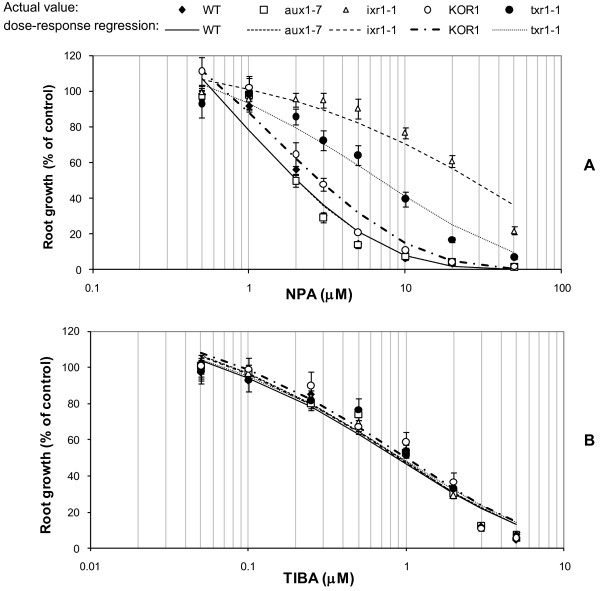
**Impact of auxin transport inhibitors on root growth of *Arabidopsis *lines.** Wild type and mutant *Arabidopsis thaliana* seedlings were treated for 72 h on medium containing: (**A**) 1-napthylphthalamic acid – NPA, or (**B**) 2,3,5-Triiodobenzoic acid - TIBA. Individual data points are expressed as mean percentages ± SE (n = 20) of control root growth on medium with no exogenous auxin transport inhibitors applied.

All three *A. thaliana* CBI mutants examined (*ixr1-1*, *txr1-1, KOR1*) showed enhanced root-based resistance to the auxin transport inhibitor, NPA, in comparison to the WT (Figure 2A). Concentration levels of NPA required to inhibit root growth by 50% (RG_50_) were approximately 13-fold (*ixr1-1*; RG_50_: 27.1 μM; *P* < 0.0001, *F* = 25.2), 3.5-fold (*txr1-1*; RG_50_: 7.08 μM; *P* = 0.0005, *F* = 14.5) and 1.5-fold (*KOR1*; 3.07 μM, *P* = 0.047, *F* = 4.2) higher than the WT (RG_50_: 2.04 μM). The response of *aux1-7* was no different to the WT (*P* = 0.94, *F* = 0.01). In contrast, all mutant lines and the WT control examined showed equivalent root growth suppression when treated with various concentrations of the auxin transport inhibitor, TIBA (Figure 2B).

### Root ion fluxes after pretreatment with auxin transport inhibitor, NPA

**Figure 3:**
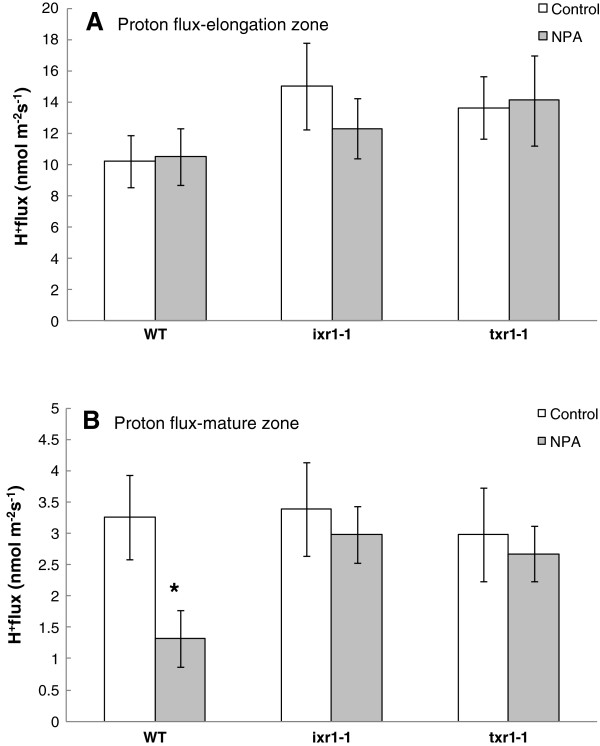
**Net H**^**+ **^**fluxes from various root zones of *Arabidopsis *lines treated with auxin transport inhibitor NPA.** Measurements were taken from the elongation (**A**) and mature (**B**) root zone of *Arabidopsis thaliana* plants after 24 h of treatment with NPA. Mean ± SE (n = 8). The flux convention is “influx positive”. * significantly different from the control of that genotype (*P < 0.05, *t* test).

No differences between *A. thaliana* genotypes were revealed in analyses of net H^+^ fluxes in the root elongation zone in response to NPA; all plants showed net H^+^ uptake of 10 to 16 nmol m^-2^ s^-1^, not significantly different to the untreated control (Figure 3A). In the mature root zone, lower net H^+^ uptake (0.5 to 5 nmol m^-2^ s^-1^) was recorded across all genotypes. In contrast to elongation zone, NPA induced a significant reduction in net H^+^ uptake, in WT (*P* < 0.05), but not in the CBI resistant mutants (*ixr1-1*, *txr1-1*) (Figure 3B).

### Mutants with differential response to CBIs

**Figure 4:**
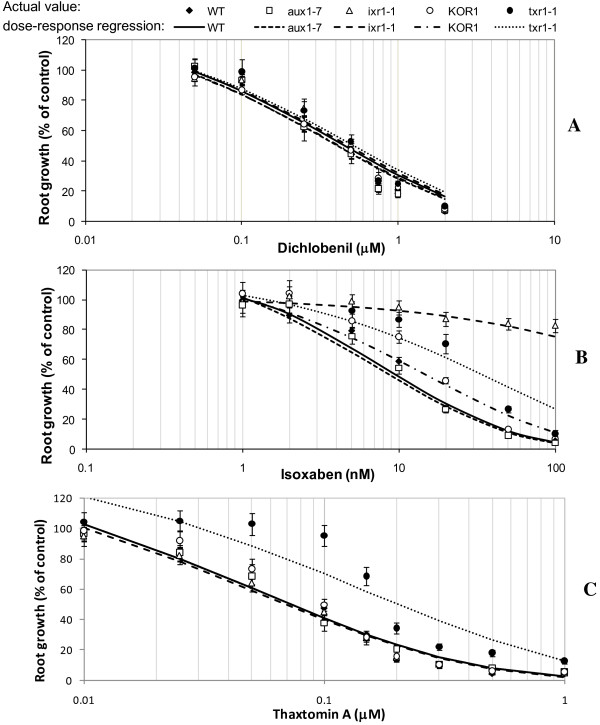
**Root growth suppression of *Arabidopsis *lines in response to cellulose biosynthetic inhibitor treatment.** Wild type and mutant *Arabidopsis thaliana* seedlings were treated for 72 h on medium containing (**A**) Dichlobenil; (**B**) Isoxaben, or (**C**) Thaxtomin A. Individual data points are expressed as mean percentages ± SE (n = 20) of control root growth on medium with no exogenous cellulose biosynthetic inhibitors applied.

Root growth rates in the *A. thaliana* lines tested varied between 5 and 9 mm day^-1^ (depending on genotype) under control conditions where no toxin was applied (data not shown). Root growth patterns in response to DCB were consistent across all five genotypes tested with RG_50_ values not significantly different compared to the control WT genotype (Figure 4A).

In response to IXB, the three CBI mutants showed enhanced tolerance with *aux1-7* responding no differently to the WT (Figure 4B). The magnitude of enhanced tolerance varied with *ixr1-1, txr1-1 and KOR1* having approximately 10-fold (*P* < 0.0001, *F* = 26.3), 4-fold (*P* = 0.0004, *F* = 15.2) and 1.8-fold (*P* = 0.04, *F* = 4.8) greater tolerance respectively to IXB, than the WT.

Root growth rates for each line decreased with increasing TA concentrations (Figure 4C), with other typical symptoms including hypertrophy, necrosis and chlorosis [15,17,24] also observed. As expected [15], in the presence of TA, the *txr1-1* mutant had a significantly greater root growth rate (RG_50_ of 0.20 μM; *P* < 0.0001, *F* = 24.5) than the WT (RG_50_ of 0.073 μM). No other mutant had TA tolerance different to the WT (*aux1-7, P* = 0.92, *F* = 0.01; *ixr1-1, P* = 0.77, *F* = 0.09, and *KOR1, P* = 0.85, *F* = 0.03) (Figure 4C).

### Quantification of CBI levels in root tissue after CBI and NPA treatment

Consistent with previous studies [15], *txr1-1* was the only mutant to accumulate significantly (*P* < 0.05, 4-fold) less TA than all other lines tested; with the addition of NPA in combination with TA producing the same result (Table 1). IXB quantification from the root tissue showed some variation although there were no significant differences produced across the lines tested with or without NPA treatment. The line *txr1-1* showed the lowest levels of detection (Table 1).

**Table 1:** **Quantification of thaxtomin A and isoxaben from *A. thaliana *root tissue**^**1**^

**A. thaliana line**	**Thaxtomin A (ng g**^**−1 **^**FW)**		**Isoxaben (ng g**^**−1 **^**FW)**	
	**−NPA**	**+NPA**	**−NPA**	**+NPA**
WT	154 ± 23^b^	135 ± 25^b^	45 ± 15	37 ± 18
aux1-7	143 ± 21^b^	126 ± 27^b^	49 ± 26	43 ± 19
ixr1-1	176 ± 26^b^	144 ± 19^b^	30 ± 14	32 ± 16
KOR1	130 ± 29^b^	112 ± 23^b^	31 ± 14	29 ± 15
txr1-1	44 ± 15^a^	40 ± 15^a^	27 ± 12	27 ± 13

^1^thaxtomin A and isoxaben levels from root tissue were quantified after 24 h on treatment media which contained either 1 μM thaxtomin A ± 3 μM NPA; or 100 nM isoxaben ± 3 μM NPA. Results are the means ± SE of three replicates.

^a, b^Statistically different values (*t*-test, P < 0.05) are indicated with a different letter in each column.

## Discussion

### The auxin: thaxtomin interaction - auxin may not be directly related with resistance to CBIs

Our previous studies with IAA and NAA in *A. thaliana*[13,17] and with 2,4-D in potato [17] demonstrated a negative association between TA toxicity and auxin content of treatment medium or concentration within plant tissues. However, the specific nature of this association is not known. Recent transcriptional profiling studies in response to both TA and IXB showed few auxin-responsive genes were upregulated by these CBIs and the authors argued that these compounds do not act on auxin receptors [12]. Additionally, Errakhi and colleagues [25] found that addition of IAA had no effect on TA induced electrophysiological H^+^ changes in *Arabidopsis*, and suggested that TA and IAA do not interact at this level. Supporting these findings we show that the root growth response of three CBI *A. thaliana* mutants (*ixr1-1*, *KOR1*, *txr1-1*) was not altered by any of the three auxins (2,4-D, IAA and NAA). This suggests that common mechanisms that confer resistance in these mutants: *ixr1-1* - altered specific cellulose synthase (CesA) complexes [8,9,26]; *KOR1* – cellulose deficiency [21,22,27]; *txr1-1* - altered cellulose synthesis activity [7,15]; does not influence auxin uptake and associated root growth response, suggesting no direct CBI – auxin interaction.

### Linkage between auxin transport and resistance to CBIs

Whilst no direct CBI – auxin interaction could be identified in this study or by others [12,25] Duval and Beaudoin [12] found that the PINOID-BINDING protein (PBP1) gene and the TOUCH3 gene (TCH3) that both bind to PINOID (PID) were upregulated in response to both IXB and TA. PID is a protein kinase that acts as a positive regulator of auxin efflux polar transport [28]. Whilst this suggested a link between inhibition of cellulose synthesis and auxin transport, more definitive proof was required [12]. Our results presented here make use of *A. thaliana* mutants which provide an important resource for delineating and understanding disease resistance pathways and mechanisms of action [19]. In this case modifications in cellulose biosynthesis, expressed in *A. thaliana* mutants, provide a link between auxin transport and inhibition of cellulose synthesis.

### Differential response of *A. thaliana* CBI mutants to two key auxin transport inhibitors

The resistance of three mutants with altered cellulose synthesis properties (*ixr1-1*, *KOR1*, *txr1-1*) to NPA implies a probable interaction between cellulose synthesis and this auxin transport efflux inhibitor. The specificity of this interaction was demonstrated by the finding that none of these mutants had an enhanced resistance to a second auxin efflux inhibitor, TIBA, that has a cellular binding site distinct to NPA [29,30].

Neither auxin efflux carrier sites nor the mode of action of inhibitors acting on these sites are fully understood [29,31] with the complexities of auxin transport and carriers constantly evolving [32-34]. However, NPA functions as a general inhibitor of secretory processes (by binding to a putative NPA-binding protein), associated with the auxin efflux carrier site [31,35]. TIBA by contrast has its binding site directly associated with the auxin efflux carrier site [30] distinct from the putative NPA-binding protein [35]. It is important to note that auxin transport and binding studies indicate that all types of auxin transport inhibitors act at a site distinct from that of the auxin binding site on the efflux carrier, i.e. they are non-competitive with auxins [30]. Taken together with the lack of any response to auxin source in all three CBI mutants, it suggests that these mutations associated with cellulose synthesis also invoke a modified response to specific compounds inhibiting polar auxin transport, i.e. NPA, rather than auxin itself, and suggests that the CBI-auxin interaction may be linked to indirect processes associated with NPA-specific binding to the auxin efflux carrier.

The NPA resistance of the *ixr1-1* and *txr1-1* mutant in our study was 13 and 3.5-fold greater than wild-type, respectively, but the nature of the NPA resistance is unknown. The finding of NPA resistance in these two CBI mutants has not previously been reported. These mutants represent a useful research tool in furthering the understanding of NPA-binding [36], cellulose biosynthesis and all its complexities.

It must be stated that the usage of NPA in these studies does have some limitations. These include the fact that NPA has a complex mode of action that is not specific for auxin transport but a general inhibitor of secretory processes [31,35]. Additionally, NPA may target different proteins and transporters at different concentrations [32,33].

### Auxin transport inhibitor, NPA affects ionic homeostasis in the mature but not elongation root zone

In the WT line, NPA treatment effects on proton flux were observed in the mature but not the elongation root zone. The suppression of H^+^ fluxes in the mature root zone mirrors the root growth suppression patterns of the three genotypes studied. The reduction in H^+^ flux may be attributed to a cessation of normal growth type responses as occurs in response to a toxin [13] or foreign compound [37]. That the major response occurred in the mature but not elongation zone was unexpected, given the well-established role of auxin in control over cell division in the root apex [38] and indicates a different mode of action. It is possible that NPA is partially suppressing the polar transport of endogenous auxin from the root apex into the mature zone, so affecting normal growth processes and flux dynamics in this mature region. In this assay, NPA tolerance was again demonstrated in CBI resistant mutants *ixr1-1* and *txr1-1*. These results further support the link between inhibition of cellulose synthesis and the auxin efflux transport processes influenced by NPA.

### Uptake of CBI’s not affected by NPA presence

To determine a possible interaction between CBI and NPA resistance the uptake of the two toxins, TA and IXB was monitored across the mutant lines. The conclusion from these quantitative assays was that NPA did not affect CBI uptake suggesting no direct interaction between these compounds. It must be stated that this work is only preliminary in nature and that the presence of auxin compounds themselves may have a greater impact on CBI uptake as this has been previously demonstrated [16,17], hence further investigation on CBI uptake with presence of auxin sources is warranted. Nonetheless, the new methodologies presented here confirm previous findings that *txr1-1* has an altered transport component that reduces TA uptake [15] and therefore less compound accumulated in the root tissue.

### Mutants with differential responses to CBIs

Testing CBI *A. thaliana* mutants as presented here or in other plant cell systems such as habituated poplar cell suspensions with resistance to CBIs [6] can aid in identifying common mechanisms across toxins [19,39] and similarities in resistance mechanisms of the mutants. Better understanding of these complexities will enable a greater understanding of how plant cells respond to toxins and key information about cellulose synthesis [6]. Indeed, similarity of responses expressed in this study particularly by *txr1-1* and *ixr1-1*, with enhanced multiple resistances to both IXB and NPA suggests that TA and IXB share a common mode of action. It is known that *txr1-1* lacks a TXR1 gene product, with the product suspected to be involved in a transport system [15]. Therefore, it is probable that both IXB and NPA may utilise this transport system in some manner. In contrast, the *ixr1-1* mutant, with an altered component of a cellulose synthase gene [9], did not show resistance to TA. The lack of cross-resistance of *ixr1-1* to TA, observed in this study suggests that this altered target enzyme is not a target of TA [12].

### TA is closely associated with IXB but more distantly with DCB

Previous research indicated a commonality in mode of action between TA and IXB, with both inhibiting cellulose synthesis [7,26], initiating a programmed cell death response [14] and upregulating a similar set of genes when applied to *A. thaliana* suspension cells [12]. Whilst we found that *txr1-1*, *ixr1-1* and *KOR1* had enhanced resistance to IXB and NPA, none of these mutants had altered resistance to DCB. Based on our complementary results from three different mutants it appears that TA is closely linked to IXB but more distantly related to DCB [12].

## Conclusions

Whilst the mode of action and specific target of TA have not yet been identified [6] data presented here are suggestive of a direct linkage between auxin efflux transport processes that may be inhibited by NPA and inhibition of cellulose biosynthesis. The MIFE ion flux data highlights an important role of the mature root zone as yet another target for possible auxin-TA interaction and provides an electrophysiological insight into these unique CBI’s. The usage of well characterized *Arabidopsis* mutants enabled associations between toxin mode of actions to be identified with IXB and TA linked, compared to the more distantly related CBI, DCB. Further work in elucidating mechanisms is required but the identification of strong resistance to NPA expressed by both *ixr1-1* and *txr1-1* makes these mutants a unique tool for further understanding auxin transport processes.

## Methods

### Plant material and chemicals

*Arabidopsis thaliana* seeds were obtained from the Arabidopsis Biological Resource Center (ABRC), Ohio State University, Columbus, Ohio USA and the Nottingham Arabidopsis Stock Centre (NASC), University of Nottingham, Loughborough, Leicestershire UK. The following mutants were selected: N298: *KOR1* – cellulose deficiency, [21,22], obtained from NASC; CS3074: *aux1-7* – mutant auxin influx carrier, resistant to NAA and 2,4-D [40,41]; CS6201: *ixr1-1* – IXB resistant [20], obtained from ABRC; and *txr1-1* – TA resistant [15], kindly supplied by R. Loria (Cornell University, Cornell, USA). All except *KOR1* have Columbia ecotype as a wild type (WT); the latter uses Wassilewkija ecotype. However, as no difference in TA responses between Columbia and Wassilewkija ecotypes was found (data not shown), only Columbia plants were used in other experiments.

TA was purified (> 98% purity) from oatmeal broth cultures of *Streptomyces scabiei* (isolate G#20) as previously described [42]. All other chemicals (including phytotoxins and hormones) were from Sigma-Aldrich Inc (St Lois, USA) unless otherwise stated. Key chemicals were dissolved in a range of solvents: TA, IXB and DCB –in methanol, IAA (Indole-3-acetic acid) and 2,4-D (2,4-Dichloro-phenoxyacetic acid) –in ethanol, NAA (1-Naphthalene acetic acid) and TIBA (2,3,5- Triiodobenzoic acid) –in NaOH, and NPA (Naptalam®: N-1-Naphthylphtalamic acid) – in Dimethyl sulfoxide (DMSO). Working solutions contained < 0.025% of these solvents and control treatments always contained the equivalent concentration of the solvent.

### Root phytotoxin assays

For all root experiments, plants were grown in a growth chamber at ambient temperature 22° ± 1°C and 16 h day length (light intensity 60 μmol m^-2^ s^-1^). *A. thaliana* seeds were surface-sterilised for 15 min in bleach solution (available Cl: 1.5% m/v). Twenty seeds were plated directly in two rows into Petri dishes containing Murashige and Skoog (MS) basal medium [43] supplemented with 8 g/L agar and 10 g/L sucrose. After a stratification period of 2 days at 4°C, plates were transferred into the growth chamber and oriented in an upright position of about 85°, enabling roots to grow along the agar surface without penetrating it. After 5 days, plants were transferred to new plates containing the MS basal medium augmented with phytotoxin and/or auxin treatments, and the position of the tip of each root was marked on the plate. After a further 3 days of incubation root length was obtained by measuring the distance the root had grown beyond the marked point, and the effect of treatment on root growth was quantified.

### CBI, auxin and auxin transport inhibitor screen

Five lines of *A. thaliana* were selected for screening (WT, *aux1-7*, *ixr1-1*, *KOR1* and *txr1-1*). Their root growth was examined on MS media augmented with (i) auxins - IAA, 2,4-D and NAA; (ii) the auxin transport inhibitors - NPA and TIBA; and (iii) the CBIs – DCB, IXB and TA. Each treatment had four replicates of five plants per plate (n = 20).

### Non-invasive ion flux measurements from A. thaliana roots

Sterilised *A. thaliana* seeds were placed on 0.8% (w/v) agar in 1.5 mL centrifuge tubes and stratified in the dark for 48 h at 4°C. The bottom of the tubes was removed and the tubes were suspended over an aerated growth solution consisting of (macronutrients) 1.25 mM KNO_3_, 0.625 mM KH_2_PO_4_, 0.5 mM MgSO_4_, 0.5 mM Ca(NO_3_)_2_ and 0.045 mM FeNaEDTA and (micronutrients) 0.16 μM CuSO_4_, 0.38 μM ZnSO_4_, 1.8 μM MnSO_4_, 45 μM H_3_BO_3_, 0.015 μM (NH_4_)6Mo_7_O_24_, and 0.01 μM CoCl_2_, adjusted to pH 5.6. The agar contained half-strength macronutrient concentrations with full strength micronutrients (pH 5.6). Genotypes (WT, *ixr1-1* and *txr1-1*) were randomly arranged under constant light at room temperature, and nutrient solutions were changed every three days. Two treatments; a control (0.025% DMSO) or 3 μM NPA (containing DMSO at a concentration of approximately 0.025%), were added to the growth medium of 11-day-old seedlings and the pH adjusted to 5.6.

Measurements of H^+^ fluxes were made after 24 h of treatment using the non-invasive ion-selective microelectrode MIFE technique (ROCU, University of Tasmania, Hobart, Australia) as described previously [13,44]. Net ion fluxes were calculated using the MIFEFLUX software for cylindrical diffusion geometry [45]. Each 12-day-old seedling was placed in a 1 ml Perspex measuring chamber containing 1 ml of the Basic Salt Medium, BSM (in mM: 0.1 KCl, 0.1 CaCl2, pH 5.6 unbuffered, plus the respective treatment), 40 min prior to measurement. Net, steady-state ion fluxes were measured for 5 min from the mature and elongation zone (1 mm and 100 μm from the root tip, respectively). Due to the effects of the treatment solutions on the performance of the ion sensors, electrodes were calibrated in solutions containing the treatment solution for that particular measurement (i.e., DMSO ± NPA), and new H^+^-selective electrodes were used for each seedling. Eight measurements were made for each treatment in both the elongation zone and the mature zone.

### Quantification of isoxaben and thaxtomin levels from *A. thaliana* roots

Genotypes were essentially grown as described above for flux measurements in a hydroponic setup. The 6-day-old seedlings were transferred to four different treatment solutions: 1 μM TA ± 3 μM NPA; 100 nM IXB ± 3 μM NPA; with all treatments containing DMSO at a concentration of approximately 0.025% and a pH of 5.6.

Plants were removed from treatment medium after 24 h, with roots excised and rinsed six times in distilled water to remove treatment solutions. Five roots were pooled together per treatment solution and genotype. They were weighed, placed in a 1.5 mL Ependorf tube, mashed with an Ependorf pestle and 600 μL of 30% methanol was added. Ceramic beads (0.5 and 2.8 mm) were added to the tubes with the tubes placed on a Vortex genie shaker for 10 min, to ensure root tissue was thoroughly macerated to enable IXB or TA to move into the methanol solution. Tubes were centrifuged at 13000 rpm for 10 min with 200 μL of the supernatant removed and stored at 4°C prior to quantification. There were three replicates of each treatment combination.

Levels of TA and IXB were determined by UPLC-MS using a Waters Acquity H-series UPLC coupled to a Waters Xevo triple quadrupole mass spectrometer. Injections were made onto a Waters Acquity UPLC C18 column (2.1 × 100 mm × 1.7 micron particles), with mobile phases A = 1% acetic acid in water and B = acetonitrile. The column was held at 35°C, the flow rate was 0.35 mL/min, and a linear gradient from 70% A, 30% B to 10% A, 90% B at 5 minutes was used, followed by a 3 minute re-equilibration between runs. Under these conditions TA eluted at 1.78 min and IXB at 4.28 min. External calibration against standard solutions was used, with a QC run after every fifth sample. Injection volume was 35 uL for thaxtomin samples and 10 uL for IXB samples.

The mass spectrometer was operated in negative ion electrospray mode for TA and positive ion electrospray mode for IXB with a switch between modes at 2.4 min. The 2 target compounds were detected by Multiple Reaction Monitoring (MRM), with channels *m/z* 437.15 to 140.0 and *m/z* 437.15 to 155.0 for TA (cone voltage 50 V for both channels, and collision energy 35 V and 27 V respectively), and *m/z* 333.15 to 150.0 and *m/z* 333.15 to 165.0 for IXB (cone voltage 27 V for both channels and collision energy 40 V and 23 V respectively). Dwell time was 145 ms per channel. The ion source temperature was 130°C, the desolvation gas was nitrogen at 950 L/hr, the desolvation temperature was 450°C and the capillary voltage was 2.8 KV.

### Data analysis

Data were subjected to analysis of variance using Genstat 11.1 (Rothamsted Experimental Station, Harpenden, Herfordshire, UK). Significance was calculated at either P = 0.05 or P = 0.01 as noted, and least significant difference (LSD) was used for comparison of treatment means. For root growth suppression data, the NLMIXED procedure in SAS (SAS/STAT, version 9.1, 2002–2003, SAS Institute Inc., Cary, NC, USA.) was used to fit the nonlinear model Y = a _*_ (b^(√(x)^) where ‘Y’ is the percentage modelled, ‘x’ is the measured variable (toxin concentration etc.), and ‘a’ and ‘b’ are parameters to be estimated for each genotype. The ‘a’ parameter corresponds to the maximum value reached at zero concentration, and ‘b’ controls the steepness of the fitted line. Comparisons of treatment effects were made by calculating the concentrations required to inhibit root growth (RG) to a 50% level, and these were compared between genotypes using F-tests; i.e. RG_50_. For MIFE ion flux data and quantification data, treatment means were separated using the *t*-test.

## Abbreviations

CBI: Cellulose biosynthetic inhibitor; CesA: Cellulose synthase; DCB: Dichlobenil; 2,4-D: 2,4-dichloro-phenoxyacetic acid; DMSO: Dimethyl sulfoxide; IAA: Indole-3-acetic acid; IXB: Isoxaben; MS: Murashige and skoog; NAA: 1-naphthalene acetic acid; NPA: 1-napthylphthalamic acid; TA: Thaxtomin A; TIBA: 2,3,5-Triiodobenzoic acid; WT: Wild-type.

## Competing interests

The authors declare that they have no competing interests.

## Authors’ contributions

RT participated in the study design, carried out the root inhibition assays, annotation and drafted the manuscript; SS participated in the design of the study and coordination; assisted with non-invasive ion flux measurements and help to draft the manuscript; TC carried out non-invasive ion flux measurements; ND developed detection methodology and carried out CBI quantification; CW conceived the study, participated in the design of the study and coordination and help to draft the manuscript. All authors read and approved the final manuscript.
